# Identifying crop research priorities based on potential economic and poverty reduction impacts: The case of cassava in Africa, Asia, and Latin America

**DOI:** 10.1371/journal.pone.0201803

**Published:** 2018-08-08

**Authors:** Arega D. Alene, Tahirou Abdoulaye, Joseph Rusike, Ricardo Labarta, Bernardo Creamer, Martha del Río, Hernan Ceballos, Luis Augusto Becerra

**Affiliations:** 1 International Institute of Tropical Agriculture (IITA), Lilongwe, Malawi; 2 International Institute of Tropical Agriculture (IITA), Ibadan, Nigeria; 3 Alliance for a Green Revolution in Africa (AGRA), Nairobi, Kenya; 4 International Centre for Tropical Agriculture (CIAT), Cali, Colombia; 5 Universidad de Las Americas (UDLA), Quito, Ecuador; Wageningen University, NETHERLANDS

## Abstract

It is widely recognized that increasing agricultural production to the levels needed to feed an expanding world population requires sharply increased public investment in research and development and widespread adoption of new technologies, but funding for national and international agricultural research has rather declined in recent years. In this situation, priority setting has become increasingly important for allocating scarce research resources among competing needs to achieve greater impacts. Using partial equilibrium economic surplus models and poverty impact simulations, this paper assesses cassava research priorities in Africa, Latin America and Caribbean, and Asia based on the potential economic and poverty reduction impacts of alternative research and technology options. The results showed that efficient planting material production and distribution systems and sustainable crop and soil fertility management practices have the greatest expected economic and poverty reduction impacts in the three regions. Lack of clean planting materials is a major constraint to adoption and it is envisaged that efficient production and distribution systems for planting material can accelerate technology adoption by farmers. Similarly, sustainable crop and soil fertility management practices play a key role in closing the observed yield gaps, especially in Africa. The paper discusses the results of the priority assessment for key cassava research options and concludes with the implications for cassava research priorities.

## Introduction

Cassava is the third most important food crop in the tropics after rice and maize and is the second most important food staple in Africa after maize accounting for more than half of the dietary calorie requirements of over 200 million people [1]. Half a billion people in Africa eat cassava every day, and this high-starch root is also an important staple in Latin America and the Caribbean. In Asia, cassava serves as a source of food and livestock feed while also providing raw material for the manufacturing of pharmaceuticals, industrial starch, biofuels, and other products [2]. As such, cassava is important not only for rural households but for national economies. Despite major biotic and abiotic threats to cassava production and productivity, cassava production has expanded especially in Africa and this is largely attributed to national and international cassava improvement research efforts [1].

International cassava improvement research was initiated in the early 1970s at the International Institute of Tropical Agriculture (IITA) and the International Center for Tropical Agriculture (CIAT) with a focus on developing high-yielding varieties with resistance to major pests and diseases [3,4]. In addition to breeding for high yield and resistance to major pests and diseases, cassava research involved developing biological control and integrated pest management options to reduce losses due to insect pests. In Sub-Saharan Africa (SSA), the work resulted in a number of several elite genotypes that had resistance to cassava mosaic disease (CMD) and cassava bacterial blight (CBB) as well as high and stable yields and good consumer acceptability. The development of improved varieties and their delivery to national programs for testing under specific local conditions during the late 1970s and 1980s has led to the successful release of high yielding and disease resistant varieties for adoption by farmers. The new varieties combine enhanced CMD tolerance with preferred postharvest characteristics, wider agroecological adaptation, and 50–100% higher yields even without the use of fertilizer [1,3].

Despite major research successes in the past, farm level cassava yields remain low especially in Africa due to a number of emerging threats such as pests and diseases. Realization of higher potential yields in farmers’ fields requires continued investment in genetic improvement and better agronomy as well as pest and disease management. To help counter the threat of pests and diseases, scientists should identify and use biotechnology tools to develop molecular markers for traits such as whitefly resistance, quantitative trait loci (QTLs) in populations derived from heterozygous parent materials, and protocols for rapid multiplication of disease-free planting materials through tissue culture.

It is widely recognized that increasing agricultural production to the levels needed to feed an increasing world population requires sharply increased public investments in research and development and widespread adoption of new technologies, but funding for national and international agricultural research has rather declined in recent years. In this situation, priority setting has become increasingly important for allocating scarce research resources among competing needs to achieve greater impacts [5]. Systematic priority assessment has been conducted since recently by combining scientists’ views on the potential for addressing particular constraints through research and technology options with an economic assessment of the benefits that could arise from adoption of those technologies [6–14]. Following its official launch in 2012, the CGIAR Research Program on Roots, Tubers and Bananas (RTB) embarked on a strategic assessment of research priorities for banana, cassava, potato, sweet potato, and yams. Using partial equilibrium economic surplus models and poverty impact simulations, this paper assesses the expected economic and poverty reduction impacts of cassava research and technology options with a view to informing strategic priority setting of cassava research in Africa, Latin America and Caribbean, and Asia. While a lot of past priority assessment work focused on strategic commodity priorities, this study undertakes crop-specific technology priority assessment. This kind of priority setting is becoming increasingly important for a number of CGIAR Research Programs (CRPs) supporting a set of priority commodities that need to focus on high-impact lines of research. The paper presents and discusses the procedures and results of the priority assessment for key cassava research options and discusses the implications for cassava research priorities.

The rest of the paper is organized as follows. The next section provides an overview of the methodology used, whereas section 3 provides details of the data sources. Section 4 presents and discusses the ex-ante impact assessment results and the last section draws conclusions and implications.

## Methods

### Economic surplus model and cost-benefit analysis

**Fig 1:**
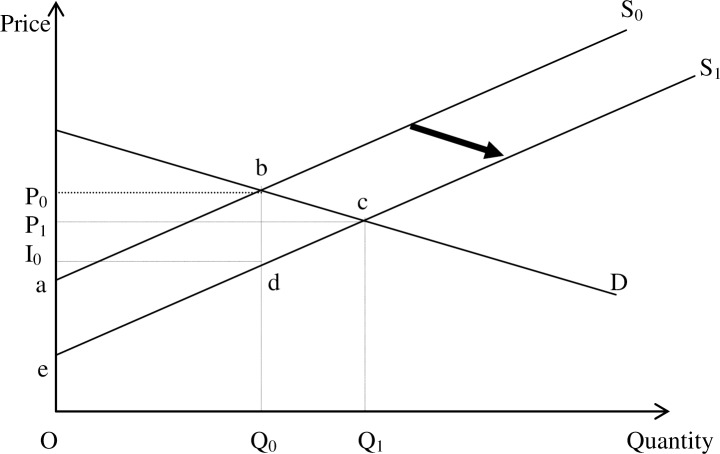
Effects of technological change on producer and consumer surplus [5].

Several impact studies of agricultural technologies have estimated aggregate economic benefits through extrapolation of farm-level yield or income gains using partial equilibrium simulation models such as the economic surplus model [5]. The economic surplus method is the most widely used procedure for economic evaluation of benefits and costs of a technological change. Technological change due to research in agriculture increases the yield, reduces yield losses, or reduces the cost of production [5]. If the new technology is yield increasing, the producer sells more of the good in the market and if demand is downward-sloping the price decreases as well. Technology adoption reduces the per-unit cost of production and hence shifts the supply function of the commodity down and to the right. If the market for the commodity is perfectly competitive, this will lead to an increase in the quantity exchanged (Q_0_ to Q_1_) and a fall in price from P_0_ to P_1_ (Fig 1). As a result, consumers benefit from the price reduction and producers may benefit from selling more of the product [5].

The economic surplus model was therefore used to derive summary measures of the potential impacts of cassava research options for a period of 25 years starting from 2014. The benefits were measured based on a parallel downward shift in the (linear) supply curve. The annual flows of gross economic benefits from cassava technologies were estimated for each of the countries and aggregated, with the aggregate benefits and costs finally discounted to derive the present value (in 2014) of total net benefits from the interventions. The key parameters that determine the magnitude of the economic benefits are: (1) the expected technology adoption in terms of area under improved technologies; (2) expected yield gains (or avoided losses) following adoption; and (3) pre-research levels of production and prices. Given the limited international trade options for cassava in most of the producing countries, the economic surplus model for the closed economy shown in Fig 1 was used to calculate the economic benefits for each country from a downward shift in the supply curve. The demand for the commodity is denoted by D, whereas the pre-research supply curve is S_0_ and the post-research supply curve following technological change is S_1_. The initial equilibrium is denoted as (P_0_, Q_0_), while the post-research equilibrium is (P_1_, Q_1_). That is, the initial equilibrium price and quantity are P_0_ and Q_0_, whereas after the supply shift they are P_1_ and Q_1_. The total benefit from the research-induced supply shift is equal to the area beneath the demand curve and between the two supply curves (ΔTS = area *abce*). The total benefit comprises the sum of benefits to consumers (ΔCS = area *P*_*0*_*bcP*_*1*_) and the benefits to producers in the form of the changes in producer surplus (ΔPS = area *P*_*1*_*ce* minus area *P*_*0*_*ba*). Under the assumption of a parallel shift (so that the vertical difference between the two curves is constant) area *I*_*0*_*de* equals area *P*_*0*_*ba*.

In a closed economy, economic surplus measures can be derived using formulas presented in Alston et al. (1995): (1) Change in economic Surplus (ΔES) = P_0_Q_0_K_*t*_(1+0.5Z_*t*_η); (2) Consumer surplus (ΔCS) = P_0_Q_0_Z_*t*_(1+0.5Z_*t*_η); and Producer Surplus (ΔPS) = (K_*t*_−Z_*t*_)P_0_Q_0_(1+0.5Zη), where K_*t*_ is the supply shift representing the product of cost reduction per ton of output as a proportion of product price (K) and technology adoption at time *t* (A_*t*_); P_0_ represents pre-research price for 2010─2012 (US$/ton); Q_0_ is pre-research level of production for 2010─2012; η is the price elasticity of demand; and Z_*t*_ is the relative reduction in price at time *t*, which is calculated as Z_*t*_ = K_*t*_ε/(ε+η), where ε is the price elasticity of supply. The research-induced supply shift parameter, K, is the single most important parameter influencing total economic surplus results from unit cost reductions and was derived as K_*t*_ = [((ΔY/Y)/ε–(ΔC/C))/(1+(ΔY/Y))]×A_*t*_ where ΔY/Y is the average proportional yield increase per hectare; ε is the elasticity of supply that is used to convert the gross production effect of research-induced yield changes to a gross unit production cost effect, ΔC/C is the average proportional change in the variable costs per hectare required to achieve the yield increase, and A_*t*_ is the rate of adoption of the improved technology at time *t*—the proportion of total cropped area under the improved varieties and practices. Annual supply shifts were then projected based on projected adoption profile for improved technologies (A_*t*_) for the period from 2014 to 2039. Adoption (A_*t*_) is assumed to follow the logistic diffusion curve.

For each country *i* (*i* = 1…*N*), the changes in economic surplus (ΔES) and the research and extension costs (C_*t*_) are discounted at a real discount rate, *r*, of 10% per annum to derive the net present values (NPV) as follows:

$$\mathrm{NPV}=\sum_{t=1}^{25}\sum_{i=1}^{N}\left(\frac{{\Delta \mathrm{ES}}_{i,}{}_{t}}{{\left(1+r\right)}^{t}}\right)-\sum_{t=1}^{25}\left(\frac{C_{t}}{{\left(1+r\right)}^{t}}\right)$$

The aggregate internal rate of return (IRR) was also calculated as the discount rate that equates the aggregate net present value (NPV) to zero as follows:

$$\sum_{t=1}^{25}\sum_{i=1}^{N}\left(\frac{{\Delta \mathrm{ES}}_{i,}{}_{t}}{{\left(1+\mathrm{IRR}\right)}^{t}}\right)-\sum_{t=1}^{25}\left(\frac{C_{t}}{{\left(1+\mathrm{IRR}\right)}^{t}}\right)=0$$

### Estimation of poverty impacts

Extending the results of the conventional economic surplus and cost-benefit analysis, the impact of each of the cassava research options on rural poverty reduction was estimated following Alene et al. [15]. It weighs the economic surplus results according to the poverty levels in each of the countries, the share of agriculture in total GDP, and the agricultural growth elasticity of poverty. The impact of each research option on rural poverty reduction was estimated by first estimating the marginal impact on poverty reduction of an increase in the value of agricultural production using poverty reduction elasticities of agricultural productivity growth. The reduction in the total number of poor was then calculated by considering the estimated economic benefits as the additional increase in agricultural production value. Thirtle et al. [16] found that a 1% growth in agricultural productivity reduces the total number of rural poor by 0.72% in Africa, 0.48% in Asia, and 0.15% in Latin America and the Caribbean (LAC). Under the assumption of constant returns to scale, a 1% growth in total factor productivity leads to a 1% growth in agricultural production. For each country, the number of poor lifted above the $1-a-day poverty line was thus derived as follows:

$$\Delta N_{p}=\underset{\mathrm{Number}\;\mathrm{of}\;\mathrm{poor}\;\mathrm{escaping}\;\mathrm{poverty}}{\underset{︸}{\underset{\mathrm{Poverty}\;\mathrm{reduction}\;\mathrm{as}\;\%\;\mathrm{of}\;\mathrm{the}\;\mathrm{poor}}{\underset{︸}{\underset{\mathrm{Gains}\;\mathrm{from}\;R\&E\;\mathrm{as}\;\%\;\mathrm{of}\;\mathrm{agricultural}\;\mathrm{production}}{\underset{︸}{\left(\frac{\Delta \mathrm{ES}}{\mathrm{Agriculture}\;\mathrm{value}\;\mathrm{added}}\times 100\%\right)}}\times \underset{\mathrm{Poverty}\;\mathrm{elasticity}}{\underset{︸}{\frac{\partial \ln\left(\frac{N_{p}}{N}\right)}{\partial \ln(Y)}}}}}\times N_{p}}}$$

where Δ*N*_*p*_ is the number of poor lifted above the poverty line, *N*_*p*_ is the total number of poor, *N* is the total population, *Y* is agricultural productivity, and ΔES is the change in economic surplus. The poverty elasticity is interpreted as the marginal impact of a 1% increase in agricultural productivity in terms of the number of poor reduced as a percentage of the total poor (*N*_*p*_), and not of the total population.

### Estimation of the number of potential beneficiaries

Data on average crop area per household and average household size were used to estimate the numbers of beneficiaries, following a procedure and dataset developed to estimate total number of RTB poor beneficiaries [17]. Data for individual countries were obtained mostly from FAO statistical database, published sources of information, or expert opinion when needed. Estimated area under two adoption scenarios (high and low adoption) was divided by the average area per household to estimate the number of adopting households, and then multiplied by household size to estimate total number of beneficiaries.

## Data sources

### Constraints analysis and identification of research options

Expert surveys and consultations were conducted between 2011 and 2013 to guide the constraints analysis and the identification and ranking of research options. Recognizing the importance of farmers’ voice in priority setting of agricultural research, a literature review was first undertaken to take stock of available evidence and secondary data on production and market constraints, technology preferences, yield gaps, and farm level impacts from baseline and adoption studies involving farmers as well as from on-farm farmer participatory research work. The outcome of the review served as a guide not only for designing the questionnaires used for the expert surveys but also for facilitating the consultations during workshops that were organized to elicit and validate individual expert opinions and estimates about the major constraints, yield gaps, and the prospects of a range of promising research and technology options. The surveys engaged stakeholders from a broad range of disciplines and backgrounds. The cassava expert community was involved in the identification of the production and market constraints and in the selection of research and technology options that can address the identified constraints. Consulting a broad range of experts with different fields of expertise enabled us to capture key constraints irrespective of institutional priorities and capacity. Overall, the expert surveys enabled the identification of the major constraints and associated research options to be included in the ex-ante impact assessment in the subsequent steps of the priority assessment exercise.

The identification of cassava research options started with analysis of the data obtained from the global expert survey in which a sample of 343 cassava experts identified the priority constraints to cassava production, processing, and marketing. The opinions of scientists who are closely involved in research on cassava production, processing, and market constraints served as the major source of information for identifying research options to address those constraints. For this objective, a global survey instrument was designed in consultation with scientists at CIAT and IITA in Spanish, English, French, and Portuguese. A global online survey of cassava experts was conducted in 2012 using the online Survey Monkey tool and 60 questionnaires were completed. In addition, questionnaires were administered to cassava experts who attended international events. A total of 282 responses were obtained at the Second Scientific Conference of the Global Cassava Partnership for the 21st Century, held on 18–22 June 2012, in Kampala, Uganda. At the 16th Triennial Symposium of the International Society for Tropical Root Crops held on 23–28 September 2012 in Abeokuta, Nigeria, another 29 questionnaires were completed. Finally, cross-country surveys of the national cassava programs and expert consultations were conducted in 2013 in Africa as well as in Latin America and the Caribbean (LAC) and Asia. The results of the survey based on the 343 completed questionnaires are presented in Alene et al. [18]

Potential research options were identified based on the expert surveys and consultations for further formal evaluation using the economic surplus model [5]. These research options included those that address the constraints relating to: (1) root yields; (2) production costs; (3) postharvest processing and utilization; and (4) sustainable production. The initial list of research options was presented and discussed with the scientists from IITA and CIAT, and later at the RTB priority assessment task force workshop held on 12–16 August 2013, in Lima, Peru. These research options were later linked with CIAT and IITA research outputs. The research options were selected to match selected research options associated with RTB flagship projects, which contribute to the required attainment of Intermediate Development Outcomes (IDOs). The final set of research options was then developed and agreed upon at the final workshop held on 12–14 November 2013 in Lima. These included: (1) High-yielding varieties with resistance to major diseases (CMD/CBSD); (2) High-yielding varieties with high dry matter and starch; (3) High-yielding varieties with longer shelf life; (4) High-yielding, drought- tolerant varieties and increased water-use efficiency; (5) Sustainable crop and soil fertility management practices; (6) Integrated pest and disease management practices, including resistant varieties; (7) Efficient and massive high-quality planting material production and distribution systems; (8) Processing technologies for value addition; (9) Strategies to prevent introduction of exotic pests and diseases; and (10) High-yielding varieties tolerant to cold weather and frost. A detailed description of the cassava research options is provided in Alene et al.[18].

### Expert estimates of the values of key parameters

Cassava research and extension experts served as the major source of information for the economic surplus analysis of cassava research options. A structured questionnaire was developed to guide consultations with IITA and CIAT scientists as well as with NARS partners in Africa, LAC, and Asia who are working on particular cassava production and market constraints to elicit key parameter estimates for the research options addressing those constraints. Expert consultations at IITA involved 12 scientists: cassava breeders (6), agronomists (3), virologists (2), and processing and utilization specialists (1). The cross-country survey in Africa involved 30 experts from NARS partners in Africa: Benin (1), Cameroon (1), DRC (1), Ghana (4), Kenya (1), Mozambique (3), Nigeria (2), Togo (3), Uganda (3), Tanzania (9), and Zambia (2). In CIAT, a group of 14 scientists (breeders, agronomists, postharvest processing experts, molecular biologists, entomologists, plant physiologists, and virologists) working in LAC and Asia was consulted. An online survey was also designed and implemented and 46 responses were obtained.

For each research option identified, scientists were asked to estimate the values of the following key parameters: maximum adoption rate, year of beginning of adoption, years to maximum adoption rate, expected yield increase (%), area affected by the constraint (%), cost change due to inputs (%), and probability of research success (%). The values of some parameters such as research costs were assembled from several sources, such as RTB program proposal and past empirical work [15,16] as well as from FAO (http://faostat.fao.org/) and the World Bank (http://data.worldbank.org/indicator). The limitation of expert opinion surveys relates to the degree of subjectivity with the estimation of the values of key parameters that determine the size of the expected benefits. While it is true that many of the judgements made in the process are subjective, the use of a more transparent, participatory and iterative approach facilitates greater dialogue and consensus building to ensure some level of objectivity. Table 1 presents the description of the key project, technology, and market-related parameters used and the corresponding data sources.

**Table 1:** Assumptions and data sources for key parameters used in the economic surplus analysis.

Parameter	Assumption/Source
Time period	25 years (2014–2039); 10 years for research investment—research lag (maximum time period for RTB). Most of the R&D investments will run for 10 years, though other research options may either be longer or shorter.
Elasticities of supply and demand	Elasticities of supply and demand were assumed to be 1 and 0.5 respectively across technologies and for all countries due to limited availability of information.
Productivity effects	Expert estimates for a particular technology supported by field trial data.
Input cost changes	Expert estimates for a particular technology supported by farm-level surveys; changes in costs for particular inputs estimated in terms of relative share in overall production costs.
Probability of research success (the probability of successfully completing the research and developing the intended technology with the desired characteristics such as higher yielding, early maturing/bulking, greater resistance to diseases, greater tolerance to drought, etc.)	Maximum value of 80% for quick wins was assumed and lower values if uncertainty of research success is higher (or implementation uncertain—e.g., GM crops). Success probabilities should be different across technologies, allowing for differences at least across regions for the same technology. Country-level success probabilities were not available, but these could be included in subsequent assessments.
Depreciation rate	1% across all technologies and countries
Discount rate	10%
Production	National average annual production for 2010–2012 from FAOSTAT (http://faostat.fao.org/). Where data were missing, we used data from previous years.
Prices	National average annual production and prices for 2009–2011 from FAOSTAT (2013). Where data were missing, we used data from previous years.
Adoption profile	Logistic adoption curve; adoption ceiling based on expert estimates (as share of total area in potential adoption domain); time to reach adoption ceiling (in years); adoption rate in first year of adoption is 1% of adoption ceiling for all technologies; year of first adoption and year of disadoption based on timeframe and expert assessment. Two adoption scenarios: (1) adoption scenario based on expert assessment and (2) conservative adoption scenario: 50% of expert assessment.
R&D and dissemination costs	Research costs estimated as the sum of: (1) RTB budgets as presented in the program proposal by thematic area (some themes actually matching the research options identified); (2) bilateral projects at IITA and CIAT (assumed to be equal to RTB budgets); and (3) NARS costs, which are assumed to be equal to IITA and CIAT budgets combined. Dissemination costs for new variety is (US$50/ha) and (US$80/ha) for other knowledge-intensive technologies, such as crop management interventions.
Poverty	Poverty incidence (% living on less than US$1.25/day), the number of poor people, and agricultural value added from World Bank’s World Development Indicators database (http://data.worldbank.org/indicator).
Agricultural value added	World Bank’s World Development Indicators database (http://data.worldbank.org/indicator).
Number of beneficiaries	Country-specific estimates prepared for RTB proposal: crop area per HH for specific crop and number of persons per HH.

### Data on socioeconomic parameters

Table 2 presents the data on the key socioeconomic parameters used in the economic surplus analysis of cassava research options for individual countries in Africa, Asia, and LAC. Data on annual harvested area, production, and producer prices were obtained from the FAOSTAT database (http://faostat.fao.org/). We used three-year national averages for each country for the period 2010–2012. In cases where FAO data were not available for particular countries and years (e.g., producer prices), we used data obtained from the respective ministries of agriculture and offices of statistics. Data on the incidence of poverty, the number of poor, and agricultural value added were obtained from the World Bank’s World Development Indicators database (http://data.worldbank.org/indicator).

**Table 2:** Data on the socioeconomic parameters used in the economic surplus analysis.

Country	Price (US$/ton)	Quantity ('000 tons)	Area harvested ('000ha)	Household size (persons)	Area per farm (ha)	Poverty incidence (%)	Number of poor (million)	Agricultural Value Added (US$ billion)
Angola	350	13,673	936	6	0.50	56	10.7	10.6
Benin	470	3,611	251	5	0.50	45	4.0	2.5
Burkina Faso	268	4	3	5	0.50	45	7.4	3.5
Burundi	374	564	65	5	0.50	81	6.8	0.9
Cameroon	357	3,744	263	5	0.50	9	1.8	4.9
Chad	698	230	22	5	0.50	45	5.0	1.5
Congo	330	1,177	135	5	0.50	53	2.2	0.5
Cote d’Ivoire	243	2,309	347	5	0.50	24	4.7	6.2
DRC	330	15,224	1,960	5	0.50	86	56.8	8.1
Ghana	163	13,325	883	4	0.50	25	6.0	9.2
Guinea	354	1,065	129	6	0.50	42	4.2	1.5
Kenya	130	608	64	4	0.50	41	16.4	11.0
Liberia	295	494	62	6	0.50	83	3.3	0.9
Madagascar	171	3,173	473	5	0.50	78	16.2	2.9
Malawi	333	4,028	194	4	0.50	67	10.0	1.3
Mozambique	201	8,501	1,267	5	0.50	60	13.9	4.4
Nigeria	259	43,920	3,449	4	0.50	68	107.2	85.9
Rwanda	299	2,325	196	4	0.50	67	7.1	2.3
Senegal	328	164	26	9	0.50	25	3.1	2.1
Sierra Leone	295	446	84	6	0.50	45	2.6	2.2
Togo	174	934	148	5	0.50	39	2.3	1.2
Uganda	120	5,073	417	5	0.50	43	14.3	4.7
Tanzania	210	5,037	898	5	0.50	67	41.5	7.8
Zambia	240	1,193	200	5	0.50	66	8.6	4.0
Argentina	116	182	18	4	0.40	1	0.4	49.1
Bolivia	299	249	29	4	0.50	16	1.6	3.1
Brazil	125	24,907	1,761	5	0.75	6	12.1	123.8
Cambodia	263	4,038	189	4	0.50	19	2.7	4.7
China	127	4,528	277	4	0.25	12	158.6	732.2
Colombia	310	2,166	204	5	0.40	8	3.8	23.5
Costa Rica	238	500	34	5	1.00	3	0.1	2.5
Cuba	62	402	71	5	1.00	2	0.2	3.0
Ecuador	245	57	19	5	1.00	5	0.7	7.8
Haiti	160	573	140	5	0.20	62	6.2	1.9
India	160	8,586	245	5	0.60	33	399.1	337.1
Indonesia	198	23,322	1,180	12	0.50	16	39.5	127.0
Jamaica	449	18	1	5	0.75	0.21	0.01	1.0
Laos	160	465	20	5	0.50	34	2.2	2.6
Malaysia	231	48	3	5	0.50	1	0.2	34.6
Paraguay	63	2,563	180	4	0.45	7	0.5	5.5
Peru	165	1,174	100	4	0.40	5	1.5	10.6
Philippines	132	2,118	218	4	0.50	18	17.5	29.2
Thailand	60	24,669	1,210	4	0.50	0.38	0.3	41.5
Venezuela	922	498	36	4	0.50	7	2.0	19.0
Vietnam	112	9,008	521	4	0.50	17	14.8	27.2

Source: FAOSTAT (http://faostat.fao.org/ and World Bank (http://data.worldbank.org/indicator).

We also used poverty elasticities of 0.72, 0.48, and 0.15 for Africa, Asia, and LAC, respectively [16]. The data on cassava area per household and household size that were used for the estimation of the numbers of beneficiaries were taken from a dataset put together for the estimation of the potential number of beneficiaries of the RTB program [17].

### Data on technology development, dissemination, and adoption parameters

The economic surplus model employed for the ex-ante impact analysis typically uses market-related data on socioeconomic parameters and technology-related data on technology development, dissemination, and adoption parameters [5]. Therefore, in addition to the socioeconomic parameters such as production and prices, the economic surplus model uses a number of parameters that relate to the research and dissemination process and includes those that relate to the expected effects of new technology adoption on yield gains (or reduced yield losses) and production costs. In addition to parameters related to expected yield gains and production cost changes following technology adoption by farmers, other technology-related parameters of importance include (1) the research lag defined as the number of years it takes until an adoptable innovation will be available to farmers; (2) adoption ceiling defined as the maximum adoption rate as a proportion of total cropped area; (3) adoption lag defined as the number of years until maximum adoption is reached; (4) the costs required to conduct R&D (i.e., R&D costs); (5) the dissemination costs for each technology (either US$80 or US$50 for every new hectare of adoption depending on the type of technology); and (6) the probability of research success.

Since the outcomes of research investments cannot be realized for many years, ex-ante technology generation and adoption parameters can only be based on the opinions of R&D experts who draw on a wealth of experience and knowledge in making informed predictions. Most of the data relating to cassava technology development, dissemination, and adoption were obtained primarily through expert surveys and consultations. Expert estimation of the values of some of these parameters involved a number of steps designed to facilitate the elicitation process. For example, estimation of the adoption ceiling involved estimation of the area affected by the underlying constraint as a proportion of the total cropped area and the expected adoption rate as a proportion of the affected area. For Africa, the affected area was thus used only to facilitate the estimation of the ultimate value of adoption as a proportion of the total cropped area. That is, adoption as a proportion of total cassava area is estimated as the product of adopting a proportion of the affected area and the affected area as a proportion of total area. For almost all research options, however, cassava experts working especially in Africa argue that much of the cassava area has been (or is expected to be) affected by the underlying constraints, such as low yield potential, poor resistance to pests and diseases, shorter shelf life, and lack of clean planting material multiplication and distribution system. Consequently, the experts argue that improved seed systems and improved varieties with high-yield attributes would be appropriate for almost all recommendation domains. However, varieties with resistance to pests and diseases should be developed not only for those areas that are currently affected by the diseases but also for all areas that will be affected in the many years to come (including pre-emptive measures). Indeed, using currently affected area as a recommendation domain for adoption would understate potential adoption of those technologies. Looking at the nature of most of our research options that make explicit mention of “high yield,” they also say that much of the cassava area should be a relevant adoption domain, especially because wider geographic adaptation is also one of the key criteria of varietal release.

On the other hand, R&D costs were estimated as the sum of (1) CRP-RTB investments in cassava research disaggregated by research theme [17]; (2) bilateral project funding for IITA (mainly for Africa) and CIAT (mainly for Asia and LAC), which was estimated to be approximately equal to the CRP-RTB funding; and (3) NARS partner costs, which were assumed to be equal to the total of CRP-RTB and bilateral funding through IITA and CIAT. Aggregating the costs across countries for each research option gives the global R&D costs needed for calculating the global NPVs and IRRs. The CRP-RTB costs were estimated based on the allocations in the RTB program proposal. The annual cassava budget was allocated across the research options. For some options such as “planting materials,” the RTB proposal had details of the allocation already made and only required little adjustment to reallocate the overheads and CRP management costs. Dissemination costs were estimated to be US$50 per hectare of adopted area for new varieties and US$80 per hectare of adopted area for other knowledge-intensive technologies, such as crop management interventions.

Table 3 provides an overview of the parameters related to cassava research and technology dissemination process. Cassava research in Africa dates back to 1936, when scientists started doing research to address major production constraints such as CMD. However, efforts to address CBSD by developing varieties with dual resistance to both CMD (including the new Uganda variant) and CBSD started recently. As can be judged from the year when research started to address particular constraints, some research and technology options have been pursued for a number of years whereas other lines of research started only recently before they were both integrated into the new RTB program (2012–14). In this assessment, we treat all past research costs as sunk costs—that is, costs excluded from the computation of research costs. Thus the information on how long the research has already been conducted puts the result of the assessment in perspective as one would expect higher NPVs and IRRs for research options with much of the R&D cost not accounted for. Clearly, the IRR measure favors such research options due to shorter research lags and higher probability of research success.

**Table 3:** Overview of parameters related to cassava research and technology dissemination process.

Technology	Year when research started		Number of countries targeted		R&D costs from 2014 (US$ million/year)			Dissemination cost (US$/ha)	
	Africa	LAC/Asia	Africa	LAC/Asia	Africa	LAC/Asia	Total	Africa	LAC/Asia
High-yielding varieties with resistance to major diseases (CMD/CBSD)	2007		24		3.88		3.88	50	
High-yielding varieties with high dry matter and starch	2007	1980	24	21	3.88	3.88	7.76	50	50
High-yielding varieties with longer shelf life	2014	2014	24	21	3.88	3.88	7.76	50	50
High-yielding, drought- tolerant varieties and increased water-use efficiency	2009	2010	24	21	3.88	3.88	7.76	50	50
Sustainable crop and soil fertility management practices	1980	1980	24	21	3.88	3.88	7.76	80	80
Integrated pest and disease management practices, including resistant varieties	1983	1998	24	21	3.88	3.88	7.76	80	80
Efficient and massive high-quality planting material production and distribution systems	2007	1995	24	21	4.39	4.39	8.78	80	80
Processing technologies for value addition	2003	2003	24	21	4.19	4.19	8.38	80	80
Strategies to prevent introduction of exotic pests and diseases		2014		21		3.88	3.88		80
High-yielding varieties tolerant to cold weather and frost		2014		21		3.88	3.88		50

### Parameter estimates for individual research options

The estimates of the parameters used in the economic surplus analysis such as maximum adoption rate, research lag, years to maximum adoption rate, percentage yield increase, cost changes due to inputs, and probability of success that are specific to each research option. This section provides an overview of the parameter estimates for each research option.

- High-yielding varieties with dual resistance to CMD and CBSD: (1) maximum adoption rate of 30–50%; (2) research lag of 5–10 years; (3) adoption lag of 12 years; (4) yield increase of 30%; (5) input cost change of 20%; and (6) probability of success of 50%.
- High-yielding varieties with high dry matter and starch: (1) maximum adoption rate of 8–90%; (2) research lag of 3–8 years; (3) adoption lag of 12 years for all African countries and 10 for all LAC and Asian countries; (4) yield increase of 15–30%; (5) input cost change of 15–20%; and (6) probability of success of 50–70%.
- High-yielding varieties with longer shelf life: (1) maximum adoption rate of 8–90%; (2) research lag of 5–8 years; (3) adoption lag of 10–14 years; (4) yield increase of 6–65%; (5) input cost change of 5–20%; and (6) probability of success of 50–80%. Expected reduction in postharvest losses as a proportion of total production following adoption of varieties with longer shelf life was taken as the yield loss avoided and was estimated as the product of (1) current postharvest losses as a proportion of total production and (2) expected reduction in postharvest losses (as a proportion of current losses) following adoption of varieties with longer shelf life.
- High-yielding, drought-tolerant varieties and increased water-use efficiency: (1) maximum adoption rate of 8–90%; (2) research lag of 5–8 years; (3) adoption lag of 12 years; (4) yield increase of 15–35%; (5) input cost change of 10–20%; and (6) probability of success of 65–80%.
- Sustainable crop and soil fertility management practices: (1) maximum adoption rates of 20–50%; (2) research lag of 1–5 years; (3) adoption lag of 8–12 years; (4) yield increase of 15–55%; (5) input cost change of 5–30%; and (6) probability of success of 75–80%. This research option generally has short research lags because of the advanced stage of development of the components of the technological packages. In view of significant yield responses of cassava to crop and soil fertility management practices, the experts also estimated a relatively higher yield increase of 15–55% as compared to the rest of the research options.
- Integrated pest and disease management practices, including resistant varieties: (1) maximum adoption rate of 8–90%; (2) research lag of 5–8 years; (3) adoption lag of 12 years; (4) yield increase of 25–70%; (5) input cost change of -30 to 20%; and (6) probability of success of 50–80%.
- Efficient and massive high-quality planting material production and distribution systems: (1) maximum adoption rate of 20–50%; (2) research lag of 1–4 years; (3) adoption lag of 5–12 years; (4) yield increase of 30–50%; (5) input cost change of 5–25%; and (6) probability of success of 50–80%. This research option has the shortest research lag of one year for many countries in LAC and Asia.
- Processing technologies for value addition: (1) maximum adoption rate 10–34%; (2) research lag of 2–8 years; (3) adoption lag of 8–12 years; (4) yield increase of 15–35%; (5) no production cost change due to inputs—that is, a postharvest technology involving no varietal change; and (6) probability of success of 50–80%. The expected yield gains were estimated indirectly based on the supply response to price increases attributable to value addition through processing. With a unitary price elasticity of supply, cassava price changes due to processing and value addition translate into equivalent production increases. As the area under cassava can be reasonably assumed to be fixed in the short run, production increases in response to price increases can only be achieved through equivalent yield increases.
- Strategies to prevent introduction of exotic pests and diseases: (1) maximum adoption rate of 10–60%; (2) research lag of 5 years; (3) adoption lag of 10 years; (4) no yield increase—that is, impact of intervention realized through production cost reductions; (5) input cost change of -35 to -10%; and (6) probability of success of 50%.
- High-yielding varieties tolerant to cold weather and frost: (1) maximum adoption rate of 10% in Colombia to 100% in Argentina; (2) research lag of 8 years; (3) adoption lag of 12 years; (4) yield increase of 20%; and (5) probability of success of 50%.

## Results of the ex-ante impact and priority assessment

The ex-ante analysis was undertaken under two alternative maximum adoption scenarios: (1) “higher adoption” scenario using adoption rates of technologies estimated by experts who are usually optimistic about the prospects of the technologies they are developing, and (2) a more conservative “lower adoption” scenario with expert estimates of adoption reduced by 50%. The summary measures of the ex-ante economic benefits of cassava technologies are presented in Table 4, whereas Table 5 presents the number of beneficiaries and poverty reduction impacts. It is worth noting that the estimated economic benefits or poverty reduction impacts for the different cassava research options cannot be aggregated. This is because the assumption underlying the strategic assessment is that the research options are mutually exclusive, with only one option pursued at a time rather than all options at the same time. The discussion in this section focuses on the results under the basic “higher adoption” scenario, but Tables 4–6 also present the results under the conservative “lower adoption” scenario for comparison. As expected, halving adoption ceiling estimates of technologies only reduces the size of expected benefits and impacts on poverty reduction, but does not alter the relative importance and impacts of the various research options. The results show that each of the cassava technologies generates large NPVs of benefits, indicating the profitability of investments in the respective cassava research options. There is considerable variation in NPVs across options ranging from US$194 million for high yielding varieties tolerant to cold weather and frost to US$16.7 billion for sustainable crop and soil fertility management practices. However, because of the substantial variation in the R&D and dissemination costs needed to generate the estimated benefits, the NPVs cannot be used to rank the research options. The IRRs are a preferred measure for ranking alternative technologies.

**Table 4:** Results of ex-ante assessment of cassava technologies—adoption ceilings and benefits.

Technology	Adoption Ceiling		All Benefits			
	Lower adoption (million ha)	Higher adoption (million ha)	Lower adoption		Higher adoption	
			NPV (US$ million)	IRR (%)	NPV (US$ million)	IRR (%)
High-yielding varieties with resistance to major diseases	2.61	5.22	1,189	57	2,408	69
High-yielding varieties with high dry matter and starch	3.73	7.47	2,143	71	4,345	89
High-yielding varieties with longer shelf life	3.70	7.40	1,167	44	2,386	53
High-yielding, drought-tolerant varieties and increased water-use efficiency	3.99	7.98	3,025	61	6,127	73
Sustainable crop and soil fertility management practices	3.27	6.54	8,284	210	16,743	301
Integrated pest and disease management practices, including resistant varieties	3.82	7.64	3,732	60	7,625	71
Efficient and massive high-quality planting material production and distribution systems	3.38	6.77	7,585	416	15,299	641
Processing technologies for value addition	2.20	4.41	3,345	120	6,768	158
Strategies to prevent introduction of exotic pests and diseases	1.18	2.36	1,529	71	3,103	86
High-yielding varieties tolerant to cold weather and frost	0.32	0.63	83	23	194	30

Source: Model estimation results.

**Table 5:** Results of ex-ante assessment of cassava technologies—beneficiaries and poverty reduction.

Technology	Number of Beneficiaries				Poverty Reduction	
	Lower adoption		Higher adoption		Lower adoption	Higher adoption
	Households (millions)	Persons (millions)	Households (millions)	Persons (millions)	Persons (millions)	Persons (millions)
High-yielding varieties with resistance to major diseases	5	24	10	48	1.00	2.01
High-yielding varieties with high dry matter and starch	7	34	15	69	1.27	2.54
High-yielding varieties with longer shelf life	8	35	15	69	0.84	1.69
High-yielding, drought-tolerant varieties and increased water-use efficiency	8	36	16	73	2.00	4.03
Sustainable crop and soil fertility management practices	6	32	13	63	2.66	5.36
Integrated pest and disease management practices, including resistant varieties	7	35	15	70	1.18	2.38
Efficient and massive high-quality planting material production and distribution systems	7	33	13	66	2.10	4.22
Processing technologies for value addition	4	23	9	45	0.92	1.85
Strategies to prevent introduction of exotic pests and diseases	2	16	5	32	0.11	0.22
High-yielding varieties tolerant to cold weather and frost	1	3	1	6	0.00	0.01

Source: Model estimation results.

**Table 6:** Regional breakdown of adoption of cassava technologies.

Technology	Africa		LAC		Asia		Total
	(million ha)	Share (%)	(million ha)	Share (%)	(million ha)	Share (%)	(million ha)
High-yielding varieties with dual resistance to CMD/CBSD	5.22	100					5.22
High-yielding varieties with high dry matter and starch	5.45	73	0.37	5	1.65	22	7.47
High-yielding varieties with longer shelf life	5.22	71	0.37	5	1.81	25	7.40
High-yielding, drought-tolerant varieties and increased water-use efficiency	5.41	68	0.92	12	1.65	21	7.98
Sustainable crop and soil fertility management practices	3.97	61	1.15	18	1.42	22	6.54
Integrated pest and disease management practices, including resistant varieties (whiteflies, CBB, super elongation, and green mites)	4.94	65	1.05	14	1.65	22	7.64
Efficient and massive high-quality planting material production and distribution systems	4.54	67	0.92	14	1.30	19	6.77
Processing technologies for value addition	2.49	57	0.75	17	1.17	27	4.41
Strategies to prevent introduction of exotic pests and diseases			0.60	25	1.76	75	2.36
High-yielding varieties tolerant to cold weather and frost			0.39	62	0.24	38	0.63

Source: Model estimation results.

The results of the ex-ante analysis further show that, even under the lower adoption scenario with expert estimates of adoption reduced by 50%, the IRRs for each of the cassava research options are much higher than the standard 10% interest rate. There is, however, considerable variation in the returns on investment across research options. For the higher adoption scenario, for example, the IRRs range from 30% for high-yielding varieties tolerant to cold weather and frost to 641% for high-quality planting material production and distribution systems. Similarly, for the lower adoption scenario, the IRRs range from 23% for high-yielding varieties tolerant to cold weather and frost to 416% for high-quality planting material production and distribution systems. The results are consistent with the fact that lack of an efficient planting material multiplication and distribution system is a major constraint to cassava production. As such, the research option addressing this constraint can have very high returns on investment by unlocking the huge potential for a cassava-planting material system that promotes large-scale adoption of improved varieties. Research in this area aims to improve quality and access to cassava planting material through rapid multiplication and mass propagation methods, alternatives for micro-stakes from disease-free stocks and on-farm management of planting material, and decentralized multiplication with improved management practices—i.e., capacity building for farmers to produce their own high-quality, clean planting material.

Table 4 also presents the estimated area on which the new technology would be adopted under both the lower and higher adoption scenarios. As per definition of the scenarios, the adoption ceiling to be reached under the lower adoption scenario is half of the area under the higher adoption scenario. The estimated adoption area is an additional indicator to be considered when making funding decisions as it translates into the likely number of beneficiaries of the new technology. Similar to the NPV results, however, the adoption ceiling information should be interpreted with caution because of the different levels of investments required for each of the research options to achieve the respective maximum adoption rates. Table 5 shows the estimated number of households and individuals who will benefit from each of the research options. These estimates are determined by the adoption ceilings and the total area under cassava in Africa, Asia, and LAC. The estimated number of beneficiaries of the various research options offers an alternative perspective of their respective potential impacts. The estimates show that up to 16 million households (or 73 million people) will benefit from the different research options. High-yielding varieties with drought tolerance and water-use efficiency, high-yielding varieties with high dry matter and starch, integrated pest and disease management practices, and high-yielding varieties with longer shelf life can reach the largest number of beneficiaries because of the largest area coverage in all the regions.

The last two columns in Table 5 show the estimated poverty reduction effects of the different research options. Although the expected impacts on poverty reduction do not account for the differing R&D and extension investments across the research options, the high and low priorities implied by the poverty reduction measure are generally consistent with those based on the economic IRR. The estimated impacts on poverty reduction range from some 100,000 people for cold weather and frost tolerance research and 220,000 people for research on prevention of introduction of exotic pests and diseases to over 4 million people for efficient planting material production and distribution system and over 5 million people for sustainable crop and soil fertility management practices. As noted earlier, sustainable crop and soil fertility management practices and efficient planting material production and distribution systems also have the highest IRR, whereas developing high-yielding varieties tolerant to cold weather and frost generates the lowest IRR of 30%. The results show that an integrated approach involving sustainable crop and soil fertility management practices and an efficient planting material production and distribution system would greatly reduce poverty among the poor cassava-growing households. The expected number of poor people lifted out of poverty depends largely on the size of the total economic benefits, national poverty rates, and region-specific elasticities of poverty reduction with respect to agricultural productivity growth.

With Africa having the highest poverty rates as well as poverty elasticity, the poverty reduction measure thus favors research options generating much of the global economic benefits that accrue to Africa. This partly explains why the two options targeting Asia and LAC only (i.e., strategies to prevent introduction of exotic pests and diseases and high-yielding varieties tolerant to cold weather and frost) have the lowest expected poverty reduction effects. The relative impacts of research options on poverty reduction thus depend not only on the total economic benefits but also on the regional shares of total economic benefits. Research options generating comparable global economic benefits may actually have different poverty reduction impacts depending on Africa’s share of the total benefits. High-yielding, drought-tolerant varieties and increased water-use efficiency have lower global economic benefits than does integrated pest and disease management, but the poverty reduction impacts are greater (over 4 million vs. 2.4 million people) because Africa accounts for much of the global economic benefits from drought tolerance.

Table 6 presents information on the regional distribution of the adoption area for the different research options. For most research options, Africa accounts for over 50% of the cassava area that will be under improved varieties when maximum adoption is reached. More specifically, Africa’s area share under improved varieties ranges from 57% for processing technologies for value addition to 73% for high-yielding varieties with high dry matter and starch and 100% for high-yielding varieties with dual resistance to the major diseases CMD and CBSD. Globally, the adoption ceilings for improved cassava technologies ranges from a little over 0.5 million ha of cassava for high-yielding varieties tolerant to cold weather and frost to nearly 8 million ha for high-yielding, drought-tolerant varieties and increased water-use efficiency.

## Conclusions and implications

Assessing research priorities based on potential impacts of alternative lines of research is critical for resource allocation efforts aimed at enhancing the impact of agricultural research in the face of declining public research budgets. This paper evaluated alternative cassava research and technology options using the traditional economic surplus measures of the benefit of research as well as the likely impacts on poverty reduction. The research options included not only those needed to remove significant constraints to crop yield but also others aimed at adding value to cassava production through new varietal traits or improved post-harvest processing. The results of the priority assessment generally show high returns to each of the cassava research options evaluated, indicating the social profitability of investments in cassava research to address a whole range of production and market constraints.

Improving the quality and supply of cassava planting material and promoting integrated crop and soil fertility management options have the largest potential economic and poverty reduction impacts. Efficient planting material production and distribution systems can go a long way in addressing the observed low adoption of improved varieties due to lack of clean planting materials. Similarly, sustainable crop and soil fertility management practices play a key role in closing the observed yield gaps, especially in Africa. Clearly, research options that lead to greater technology adoption and increased root yields should have greater economic and poverty reduction impacts. The relative impacts of research options on poverty reduction depend not only on the total economic benefits but also on the regional shares of total economic benefits. As both poverty rates and poverty reduction elasticities are the highest in Africa, research options generating comparable global economic benefits may actually have different poverty reduction impacts depending on Africa’s share of the total benefits. For example, high-yielding, drought-tolerant varieties and increased water-use efficiency have lower global economic benefits than does integrated pest and disease management. However, the poverty reduction impacts are greater because Africa accounts for much of the global economic benefits from drought tolerance. The regional distribution of the adoption area for most research options shows that Africa accounts for over 50% of the cassava area that will be under improved varieties when maximum adoption is reached.

It is worth noting that for research options such as processing for value addition or varieties with longer shelf life that generate economic benefits mainly through demand shifts rather than supply shifts, there is need for further refinement of the models to fully account for economic gains due to shifts in the demand function and the resulting price changes. For cassava processing and other value addition technologies, for example, the economic surplus model used in this paper only captures the economic benefits associated with increased productivity and supply in response to higher derived demand—i.e. demand shift for processed cassava also leading to demand shift for fresh roots—and market opportunity for fresh cassava roots. As the model does not account for the more direct benefits associated with the demand shift and the value-added farmers earn from selling the processed product, there is need to develop and apply a unified framework involving both demand and supply shifts to measure the direct and indirect economic benefits associated with processing technologies for value addition.

## Supporting information

S1 Appendix Dataset for assessing cassava research priorities. (DOCX)
