# Supplementary material for: Identifying crop research priorities based on potential economic and poverty reduction impacts: The case of cassava in Africa, Asia, and Latin America
Source: PLoS One. 2018 Aug 8;13(8):e0201803. doi: 10.1371/journal.pone.0201803 (PMC6082557; doi:10.1371/journal.pone.0201803)
Supplement: S1 Appendix — (DOCX) [file pone.0201803.s001.docx]

**Identifying crop research priorities based on potential economic and poverty reduction impacts: the case of cassava in Africa, Asia, and Latin America**

# Values of technology-related parameters for individual research options

Table 1: High-yielding varieties with dual resistance to CMD/CBSD

| **Country** | **Maximum Adoption Rate** | **Research Lag**  **(years)** | **Adoption Lag**  **(years)** | **Yield Increase** | **Input Cost Change** | **Probability of Success** |
| --- | --- | --- | --- | --- | --- | --- |
| Angola | 30% | 10.00 | 12.00 | 30% | 20% | 50% |
| Benin | 40% | 7.00 | 12.00 | 30% | 20% | 50% |
| Burkina Faso | 30% | 10.00 | 12.00 | 30% | 20% | 50% |
| Burundi | 30% | 10.00 | 12.00 | 30% | 20% | 50% |
| Cameroon | 40% | 7.00 | 12.00 | 30% | 20% | 50% |
| Chad | 30% | 10.00 | 12.00 | 30% | 20% | 50% |
| Congo | 30% | 10.00 | 12.00 | 30% | 20% | 50% |
| Cote d’Ivoire | 30% | 10.00 | 12.00 | 30% | 20% | 50% |
| DRC | 40% | 7.00 | 12.00 | 30% | 20% | 50% |
| Ghana | 50% | 5.00 | 12.00 | 30% | 20% | 50% |
| Guinea | 30% | 10.00 | 12.00 | 30% | 20% | 50% |
| Kenya | 40% | 7.00 | 12.00 | 30% | 20% | 50% |
| Liberia | 30% | 10.00 | 12.00 | 30% | 20% | 50% |
| Madagascar | 30% | 10.00 | 12.00 | 30% | 20% | 50% |
| Malawi | 40% | 7.00 | 12.00 | 30% | 20% | 50% |
| Mozambique | 40% | 7.00 | 12.00 | 30% | 20% | 50% |
| Nigeria | 50% | 5.00 | 12.00 | 30% | 20% | 50% |
| Rwanda | 30% | 10.00 | 12.00 | 30% | 20% | 50% |
| Senegal | 30% | 10.00 | 12.00 | 30% | 20% | 50% |
| Sierra Leone | 30% | 10.00 | 12.00 | 30% | 20% | 50% |
| Togo | 40% | 7.00 | 12.00 | 30% | 20% | 50% |
| Uganda | 50% | 5.00 | 12.00 | 30% | 20% | 50% |
| Tanzania | 40% | 7.00 | 12.00 | 30% | 20% | 50% |
| Zambia | 40% | 7.00 | 12.00 | 30% | 20% | 50% |

**Source**: Expert consultations with IITA and NARS scientists in Africa.

Table 2: High-yielding varieties with high dry matter and starch

| **Country** | **Maximum Adoption Rate** | **Research Lag**  **(years)** | **Adoption Lag**  **(years)** | **Yield Increase** | **Input Cost Change** | **Probability of Success** |
| --- | --- | --- | --- | --- | --- | --- |
| Angola | 30% | 8.00 | 12.00 | 30% | 20% | 50% |
| Benin | 40% | 5.00 | 12.00 | 30% | 20% | 50% |
| Burkina Faso | 30% | 8.00 | 12.00 | 30% | 20% | 50% |
| Burundi | 30% | 8.00 | 12.00 | 30% | 20% | 50% |
| Cameroon | 40% | 5.00 | 12.00 | 30% | 20% | 50% |
| Chad | 30% | 8.00 | 12.00 | 30% | 20% | 50% |
| Congo | 30% | 8.00 | 12.00 | 30% | 20% | 50% |
| Cote d’Ivoire | 40% | 5.00 | 12.00 | 30% | 20% | 50% |
| DRC | 50% | 3.00 | 12.00 | 30% | 20% | 50% |
| Ghana | 50% | 3.00 | 12.00 | 30% | 20% | 50% |
| Guinea | 30% | 8.00 | 12.00 | 30% | 20% | 50% |
| Kenya | 40% | 5.00 | 12.00 | 30% | 20% | 50% |
| Liberia | 30% | 8.00 | 12.00 | 30% | 20% | 50% |
| Madagascar | 30% | 8.00 | 12.00 | 30% | 20% | 50% |
| Malawi | 40% | 5.00 | 12.00 | 30% | 20% | 50% |
| Mozambique | 40% | 5.00 | 12.00 | 30% | 20% | 50% |
| Nigeria | 50% | 3.00 | 12.00 | 30% | 20% | 50% |
| Rwanda | 30% | 8.00 | 12.00 | 30% | 20% | 50% |
| Senegal | 30% | 8.00 | 12.00 | 30% | 20% | 50% |
| Sierra Leone | 30% | 8.00 | 12.00 | 30% | 20% | 50% |
| Togo | 40% | 5.00 | 12.00 | 30% | 20% | 50% |
| Uganda | 50% | 3.00 | 12.00 | 30% | 20% | 50% |
| Tanzania | 40% | 5.00 | 12.00 | 30% | 20% | 50% |
| Zambia | 40% | 5.00 | 12.00 | 30% | 20% | 50% |
| Argentina | 28% | 4.00 | 10.00 | 22% | 15% | 70% |
| Bolivia | 19% | 4.00 | 10.00 | 22% | 15% | 70% |
| Brazil | 10% | 4.00 | 10.00 | 22% | 15% | 70% |
| Cambodia | 68% | 4.00 | 10.00 | 22% | 15% | 70% |
| China | 8% | 4.00 | 10.00 | 22% | 15% | 70% |
| Colombia | 30% | 4.00 | 10.00 | 22% | 15% | 70% |
| Costa Rica | 20% | 4.00 | 10.00 | 22% | 15% | 70% |
| Cuba | 19% | 4.00 | 10.00 | 22% | 15% | 70% |
| Ecuador | 23% | 4.00 | 10.00 | 22% | 15% | 70% |
| Haiti | 15% | 4.00 | 10.00 | 22% | 15% | 70% |
| India | 33% | 4.00 | 10.00 | 22% | 15% | 70% |
| Indonesia | 8% | 4.00 | 10.00 | 22% | 15% | 70% |
| Jamaica | 17% | 4.00 | 10.00 | 22% | 15% | 70% |
| Laos | 80% | 4.00 | 10.00 | 22% | 15% | 70% |
| Malaysia | 26% | 4.00 | 10.00 | 22% | 15% | 70% |
| Paraguay | 21% | 4.00 | 10.00 | 22% | 15% | 70% |
| Peru | 26% | 4.00 | 10.00 | 22% | 15% | 70% |
| Philippines | 10% | 4.00 | 10.00 | 22% | 15% | 70% |
| Thailand | 90% | 4.00 | 10.00 | 22% | 15% | 70% |
| Venezuela | 26% | 4.00 | 10.00 | 22% | 15% | 70% |
| Vietnam | 38% | 4.00 | 10.00 | 22% | 15% | 70% |

**Source**: Expert consultations with IITA, CIAT, and NARS scientists in Africa, LAC, and Asia.

Table 3: High-yielding varieties with longer shelf life

| **Country** | **Maximum Adoption Rate** | **Research Lag**  **(years)** | **Adoption Lag**  **(years)** | **Yield Increase** | **Input Cost Change** | **Probability of Success** |
| --- | --- | --- | --- | --- | --- | --- |
| Angola | 30% | 8.00 | 12.00 | 32% | 20% | 50% |
| Benin | 40% | 7.00 | 12.00 | 28% | 20% | 50% |
| Burkina Faso | 30% | 8.00 | 12.00 | 32% | 20% | 50% |
| Burundi | 30% | 8.00 | 12.00 | 32% | 20% | 50% |
| Cameroon | 40% | 7.00 | 12.00 | 28% | 20% | 50% |
| Chad | 30% | 8.00 | 12.00 | 32% | 20% | 50% |
| Congo | 30% | 8.00 | 12.00 | 32% | 20% | 50% |
| Cote d’Ivoire | 30% | 8.00 | 12.00 | 32% | 20% | 50% |
| DRC | 40% | 7.00 | 12.00 | 28% | 20% | 50% |
| Ghana | 50% | 5.00 | 12.00 | 24% | 20% | 50% |
| Guinea | 30% | 8.00 | 12.00 | 32% | 20% | 50% |
| Kenya | 40% | 7.00 | 12.00 | 28% | 20% | 50% |
| Liberia | 30% | 8.00 | 12.00 | 32% | 20% | 50% |
| Madagascar | 30% | 8.00 | 12.00 | 32% | 20% | 50% |
| Malawi | 40% | 7.00 | 12.00 | 28% | 20% | 50% |
| Mozambique | 40% | 7.00 | 12.00 | 28% | 20% | 50% |
| Nigeria | 50% | 5.00 | 12.00 | 24% | 20% | 50% |
| Rwanda | 30% | 8.00 | 12.00 | 32% | 20% | 50% |
| Senegal | 30% | 8.00 | 12.00 | 32% | 20% | 50% |
| Sierra Leone | 30% | 8.00 | 12.00 | 32% | 20% | 50% |
| Togo | 40% | 7.00 | 12.00 | 28% | 20% | 50% |
| Uganda | 50% | 5.00 | 12.00 | 24% | 20% | 50% |
| Tanzania | 40% | 7.00 | 12.00 | 28% | 20% | 50% |
| Zambia | 40% | 7.00 | 12.00 | 28% | 20% | 50% |
| Argentina | 28% | 8.00 | 14.00 | 22% | 5% | 80% |
| Bolivia | 19% | 8.00 | 14.00 | 30% | 5% | 80% |
| Brazil | 10% | 8.00 | 14.00 | 31% | 5% | 80% |
| Cambodia | 68% | 8.00 | 10.00 | 8% | 5% | 80% |
| China | 70% | 8.00 | 10.00 | 6% | 5% | 80% |
| Colombia | 30% | 8.00 | 14.00 | 12% | 5% | 80% |
| Costa Rica | 20% | 8.00 | 14.00 | 16% | 5% | 80% |
| Cuba | 19% | 8.00 | 14.00 | 23% | 5% | 80% |
| Ecuador | 23% | 8.00 | 14.00 | 18% | 5% | 80% |
| Haiti | 15% | 8.00 | 14.00 | 65% | 5% | 80% |
| India | 33% | 8.00 | 10.00 | 11% | 5% | 80% |
| Indonesia | 8% | 8.00 | 10.00 | 41% | 5% | 80% |
| Jamaica | 17% | 8.00 | 14.00 | 31% | 5% | 80% |
| Laos | 30% | 8.00 | 10.00 | 16% | 5% | 80% |
| Malaysia | 26% | 8.00 | 10.00 | 13% | 5% | 80% |
| Paraguay | 21% | 8.00 | 14.00 | 34% | 5% | 80% |
| Peru | 26% | 8.00 | 14.00 | 19% | 5% | 80% |
| Philippines | 10% | 8.00 | 10.00 | 49% | 5% | 80% |
| Thailand | 90% | 8.00 | 10.00 | 6% | 5% | 80% |
| Venezuela | 26% | 8.00 | 14.00 | 31% | 5% | 80% |
| Vietnam | 38% | 8.00 | 10.00 | 16% | 5% | 80% |

**Source**: Expert consultations with IITA, CIAT, and NARS scientists in Africa, LAC, and Asia.

Table 4: High-yielding, drought-tolerant varieties and increased water-use efficiency

| **Country** | **Maximum Adoption Rate** | **Research Lag**  **(years)** | **Adoption Lag (years)** | **Yield Increase** | **Input Cost Change** | **Probability of Success** |
| --- | --- | --- | --- | --- | --- | --- |
| Angola | 30% | 8.00 | 12.00 | 35% | 20% | 65% |
| Benin | 40% | 7.00 | 12.00 | 35% | 20% | 65% |
| Burkina Faso | 30% | 8.00 | 12.00 | 35% | 20% | 65% |
| Burundi | 30% | 8.00 | 12.00 | 35% | 20% | 65% |
| Cameroon | 40% | 7.00 | 12.00 | 35% | 20% | 65% |
| Chad | 30% | 8.00 | 12.00 | 35% | 20% | 65% |
| Congo | 30% | 8.00 | 12.00 | 35% | 20% | 65% |
| Cote d’Ivoire | 30% | 8.00 | 12.00 | 35% | 20% | 65% |
| DRC | 50% | 5.00 | 12.00 | 35% | 20% | 65% |
| Ghana | 50% | 5.00 | 12.00 | 35% | 20% | 65% |
| Guinea | 30% | 8.00 | 12.00 | 35% | 20% | 65% |
| Kenya | 40% | 7.00 | 12.00 | 35% | 20% | 65% |
| Liberia | 30% | 8.00 | 12.00 | 35% | 20% | 65% |
| Madagascar | 30% | 8.00 | 12.00 | 35% | 20% | 65% |
| Malawi | 40% | 7.00 | 12.00 | 35% | 20% | 65% |
| Mozambique | 40% | 7.00 | 12.00 | 35% | 20% | 65% |
| Nigeria | 50% | 5.00 | 12.00 | 35% | 20% | 65% |
| Rwanda | 30% | 8.00 | 12.00 | 35% | 20% | 65% |
| Senegal | 30% | 8.00 | 12.00 | 35% | 20% | 65% |
| Sierra Leone | 30% | 8.00 | 12.00 | 35% | 20% | 65% |
| Togo | 40% | 7.00 | 12.00 | 35% | 20% | 65% |
| Uganda | 50% | 5.00 | 12.00 | 35% | 20% | 65% |
| Tanzania | 40% | 7.00 | 12.00 | 35% | 20% | 65% |
| Zambia | 40% | 7.00 | 12.00 | 35% | 20% | 65% |
| Argentina | 30% | 8.00 | 12.00 | 25% | 10% | 80% |
| Bolivia | 30% | 8.00 | 12.00 | 25% | 10% | 80% |
| Brazil | 35% | 8.00 | 12.00 | 25% | 10% | 80% |
| Cambodia | 68% | 8.00 | 12.00 | 25% | 10% | 80% |
| China | 8% | 8.00 | 12.00 | 25% | 10% | 80% |
| Colombia | 40% | 8.00 | 12.00 | 25% | 10% | 80% |
| Costa Rica | 30% | 8.00 | 12.00 | 25% | 10% | 80% |
| Cuba | 40% | 8.00 | 12.00 | 25% | 10% | 80% |
| Ecuador | 40% | 8.00 | 12.00 | 30% | 10% | 80% |
| Haiti | 40% | 8.00 | 12.00 | 25% | 10% | 80% |
| India | 33% | 8.00 | 12.00 | 15% | 10% | 80% |
| Indonesia | 8% | 8.00 | 12.00 | 25% | 10% | 80% |
| Jamaica | 30% | 8.00 | 12.00 | 25% | 10% | 80% |
| Laos | 80% | 8.00 | 12.00 | 25% | 10% | 80% |
| Malaysia | 30% | 8.00 | 12.00 | 25% | 10% | 80% |
| Paraguay | 30% | 8.00 | 12.00 | 25% | 10% | 80% |
| Peru | 40% | 8.00 | 12.00 | 25% | 10% | 80% |
| Philippines | 10% | 8.00 | 12.00 | 25% | 10% | 80% |
| Thailand | 90% | 8.00 | 12.00 | 25% | 10% | 80% |
| Venezuela | 30% | 8.00 | 12.00 | 25% | 10% | 80% |
| Vietnam | 38% | 8.00 | 12.00 | 25% | 10% | 80% |

**Source**: Expert consultations with IITA, CIAT, and NARS scientists in Africa, LAC, and Asia.

Table 5: Sustainable crop and soil fertility management practices

| **Country** | **Maximum Adoption Rate** | **Research Lag**  **(years)** | **Adoption Lag**  **(years)** | **Yield Increase** | **Input Cost Change** | **Probability of Success** |
| --- | --- | --- | --- | --- | --- | --- |
| Angola | 20% | 5.00 | 12.00 | 50% | 25% | 75% |
| Benin | 30% | 4.00 | 12.00 | 50% | 25% | 75% |
| Burkina Faso | 20% | 5.00 | 12.00 | 50% | 25% | 75% |
| Burundi | 20% | 5.00 | 12.00 | 50% | 25% | 75% |
| Cameroon | 30% | 4.00 | 12.00 | 50% | 25% | 75% |
| Chad | 20% | 5.00 | 12.00 | 50% | 25% | 75% |
| Congo | 20% | 5.00 | 12.00 | 50% | 25% | 75% |
| Cote d’Ivoire | 20% | 5.00 | 12.00 | 50% | 25% | 75% |
| DRC | 30% | 4.00 | 12.00 | 50% | 25% | 75% |
| Ghana | 40% | 3.00 | 12.00 | 50% | 25% | 75% |
| Guinea | 20% | 5.00 | 12.00 | 50% | 25% | 75% |
| Kenya | 30% | 4.00 | 12.00 | 50% | 25% | 75% |
| Liberia | 20% | 5.00 | 12.00 | 50% | 25% | 75% |
| Madagascar | 20% | 5.00 | 12.00 | 50% | 25% | 75% |
| Malawi | 30% | 4.00 | 12.00 | 50% | 25% | 75% |
| Mozambique | 30% | 4.00 | 12.00 | 50% | 25% | 75% |
| Nigeria | 40% | 3.00 | 12.00 | 50% | 25% | 75% |
| Rwanda | 20% | 5.00 | 12.00 | 50% | 25% | 75% |
| Senegal | 20% | 5.00 | 12.00 | 50% | 25% | 75% |
| Sierra Leone | 20% | 5.00 | 12.00 | 50% | 25% | 75% |
| Togo | 30% | 4.00 | 12.00 | 50% | 25% | 75% |
| Uganda | 40% | 3.00 | 12.00 | 50% | 25% | 75% |
| Tanzania | 30% | 4.00 | 12.00 | 50% | 25% | 75% |
| Zambia | 30% | 4.00 | 12.00 | 50% | 25% | 75% |
| Argentina | 30% | 1.00 | 8.00 | 55% | 20% | 80% |
| Bolivia | 30% | 1.00 | 8.00 | 55% | 20% | 80% |
| Brazil | 50% | 2.00 | 8.00 | 56% | 20% | 80% |
| Cambodia | 30% | 1.00 | 8.00 | 50% | 20% | 80% |
| China | 50% | 1.00 | 8.00 | 40% | 20% | 80% |
| Colombia | 45% | 4.00 | 9.00 | 20% | 15% | 80% |
| Costa Rica | 40% | 1.00 | 8.00 | 50% | 20% | 80% |
| Cuba | 30% | 1.00 | 8.00 | 33% | 20% | 80% |
| Ecuador | 30% | 1.00 | 8.00 | 49% | 20% | 80% |
| Haiti | 20% | 3.00 | 6.00 | 30% | 30% | 80% |
| India | 30% | 1.00 | 8.00 | 17% | 5% | 80% |
| Indonesia | 30% | 1.00 | 8.00 | 50% | 20% | 80% |
| Jamaica | 30% | 1.00 | 10.00 | 43% | 20% | 80% |
| Laos | 30% | 1.00 | 10.00 | 50% | 20% | 80% |
| Malaysia | 30% | 1.00 | 8.00 | 50% | 20% | 80% |
| Paraguay | 30% | 1.00 | 8.00 | 55% | 20% | 80% |
| Peru | 30% | 1.00 | 8.00 | 49% | 20% | 80% |
| Philippines | 30% | 1.00 | 8.00 | 30% | 20% | 80% |
| Thailand | 43% | 1.00 | 8.00 | 15% | 10% | 80% |
| Venezuela | 30% | 1.00 | 8.00 | 20% | 5% | 80% |
| Vietnam | 40% | 1.00 | 8.00 | 50% | 20% | 80% |

**Source**: Expert consultations with IITA, CIAT, and NARS scientists in Africa, LAC, and Asia.

Table 6: Integrated pest and disease management practices, including resistant varieties

| **Country** | **Maximum Adoption Rate** | **Research Lag**  **(years)** | **Adoption Lag**  **(years)** | **Yield Increase** | **Input Cost Change** | **Probability of Success** |
| --- | --- | --- | --- | --- | --- | --- |
| Angola | 40% | 5.00 | 12.00 | 25% | 20% | 80% |
| Benin | 40% | 5.00 | 12.00 | 25% | 20% | 80% |
| Burkina Faso | 40% | 5.00 | 12.00 | 25% | 20% | 80% |
| Burundi | 40% | 5.00 | 12.00 | 25% | 20% | 80% |
| Cameroon | 40% | 5.00 | 12.00 | 25% | 20% | 80% |
| Chad | 40% | 5.00 | 12.00 | 25% | 20% | 80% |
| Congo | 40% | 5.00 | 12.00 | 25% | 20% | 80% |
| Cote d’Ivoire | 40% | 5.00 | 12.00 | 25% | 20% | 80% |
| DRC | 40% | 5.00 | 12.00 | 25% | 20% | 80% |
| Ghana | 40% | 5.00 | 12.00 | 25% | 20% | 80% |
| Guinea | 40% | 5.00 | 12.00 | 25% | 20% | 80% |
| Kenya | 40% | 5.00 | 12.00 | 25% | 20% | 80% |
| Liberia | 40% | 5.00 | 12.00 | 25% | 20% | 80% |
| Madagascar | 40% | 5.00 | 12.00 | 25% | 20% | 80% |
| Malawi | 40% | 5.00 | 12.00 | 25% | 20% | 80% |
| Mozambique | 40% | 5.00 | 12.00 | 25% | 20% | 80% |
| Nigeria | 40% | 5.00 | 12.00 | 25% | 20% | 80% |
| Rwanda | 40% | 5.00 | 12.00 | 25% | 20% | 80% |
| Senegal | 40% | 5.00 | 12.00 | 25% | 20% | 80% |
| Sierra Leone | 40% | 5.00 | 12.00 | 25% | 20% | 80% |
| Togo | 40% | 5.00 | 12.00 | 25% | 20% | 80% |
| Uganda | 40% | 5.00 | 12.00 | 25% | 20% | 80% |
| Tanzania | 40% | 5.00 | 12.00 | 25% | 20% | 80% |
| Zambia | 40% | 5.00 | 12.00 | 25% | 20% | 80% |
| Argentina | 20% | 8.00 | 12.00 | 37% | -15% | 70% |
| Bolivia | 30% | 8.00 | 12.00 | 37% | -30% | 70% |
| Brazil | 40% | 8.00 | 12.00 | 37% | -23% | 50% |
| Cambodia | 68% | 8.00 | 12.00 | 70% | -15% | 70% |
| China | 8% | 8.00 | 12.00 | 70% | -15% | 70% |
| Colombia | 40% | 8.00 | 12.00 | 37% | -30% | 50% |
| Costa Rica | 30% | 8.00 | 12.00 | 37% | -30% | 70% |
| Cuba | 30% | 8.00 | 12.00 | 37% | -30% | 70% |
| Ecuador | 30% | 8.00 | 12.00 | 37% | -30% | 70% |
| Haiti | 30% | 8.00 | 12.00 | 37% | -30% | 70% |
| India | 33% | 8.00 | 12.00 | 70% | -30% | 70% |
| Indonesia | 8% | 8.00 | 12.00 | 70% | -30% | 70% |
| Jamaica | 10% | 8.00 | 12.00 | 37% | -15% | 70% |
| Laos | 80% | 8.00 | 12.00 | 70% | -15% | 70% |
| Malaysia | 10% | 8.00 | 12.00 | 70% | -15% | 70% |
| Paraguay | 40% | 8.00 | 12.00 | 37% | -30% | 50% |
| Peru | 40% | 8.00 | 12.00 | 37% | -30% | 50% |
| Philippines | 10% | 8.00 | 12.00 | 70% | -15% | 70% |
| Thailand | 90% | 8.00 | 12.00 | 70% | -30% | 70% |
| Venezuela | 30% | 8.00 | 12.00 | 37% | -30% | 70% |
| Vietnam | 38% | 8.00 | 12.00 | 70% | -15% | 70% |

**Source**: Expert consultations with IITA, CIAT, and NARS scientists in Africa, LAC, and Asia.

Table 7: Efficient and massive high-quality planting material production and distribution systems

| **Country** | **Maximum Adoption Rate** | **Research Lag**  **(years)** | **Adoption Lag**  **(years)** | **Yield Increase** | **Input Cost Change** | **Probability of Success** |
| --- | --- | --- | --- | --- | --- | --- |
| Angola | 20% | 4.00 | 12.00 | 50% | 25% | 50% |
| Benin | 30% | 3.00 | 12.00 | 50% | 25% | 50% |
| Burkina Faso | 20% | 4.00 | 12.00 | 50% | 25% | 50% |
| Burundi | 20% | 4.00 | 12.00 | 50% | 25% | 50% |
| Cameroon | 30% | 3.00 | 12.00 | 50% | 25% | 50% |
| Chad | 20% | 4.00 | 12.00 | 50% | 25% | 50% |
| Congo | 20% | 4.00 | 12.00 | 50% | 25% | 50% |
| Cote d’Ivoire | 30% | 3.00 | 12.00 | 50% | 25% | 50% |
| DRC | 40% | 2.00 | 12.00 | 50% | 25% | 50% |
| Ghana | 40% | 2.00 | 12.00 | 50% | 25% | 50% |
| Guinea | 20% | 4.00 | 12.00 | 50% | 25% | 50% |
| Kenya | 30% | 3.00 | 12.00 | 50% | 25% | 50% |
| Liberia | 20% | 4.00 | 12.00 | 50% | 25% | 50% |
| Madagascar | 20% | 4.00 | 12.00 | 50% | 25% | 50% |
| Malawi | 30% | 3.00 | 12.00 | 50% | 25% | 50% |
| Mozambique | 30% | 3.00 | 12.00 | 50% | 25% | 50% |
| Nigeria | 50% | 2.00 | 12.00 | 50% | 25% | 50% |
| Rwanda | 20% | 4.00 | 12.00 | 50% | 25% | 50% |
| Senegal | 20% | 4.00 | 12.00 | 50% | 25% | 50% |
| Sierra Leone | 20% | 4.00 | 12.00 | 50% | 25% | 50% |
| Togo | 30% | 3.00 | 12.00 | 50% | 25% | 50% |
| Uganda | 40% | 2.00 | 12.00 | 50% | 25% | 50% |
| Tanzania | 30% | 3.00 | 12.00 | 50% | 25% | 50% |
| Zambia | 30% | 3.00 | 12.00 | 50% | 25% | 50% |
| Argentina | 30% | 1.00 | 5.00 | 30% | 5% | 80% |
| Bolivia | 30% | 1.00 | 5.00 | 30% | 5% | 80% |
| Brazil | 35% | 1.00 | 5.00 | 30% | 5% | 80% |
| Cambodia | 30% | 1.00 | 5.00 | 30% | 5% | 80% |
| China | 30% | 1.00 | 5.00 | 30% | 5% | 80% |
| Colombia | 40% | 1.00 | 5.00 | 30% | 5% | 80% |
| Costa Rica | 30% | 1.00 | 5.00 | 30% | 5% | 80% |
| Cuba | 40% | 1.00 | 5.00 | 30% | 5% | 80% |
| Ecuador | 40% | 1.00 | 5.00 | 30% | 5% | 80% |
| Haiti | 40% | 1.00 | 5.00 | 30% | 5% | 80% |
| India | 40% | 1.00 | 5.00 | 30% | 5% | 80% |
| Indonesia | 30% | 1.00 | 5.00 | 30% | 5% | 80% |
| Jamaica | 30% | 1.00 | 5.00 | 30% | 5% | 80% |
| Laos | 30% | 1.00 | 5.00 | 30% | 5% | 80% |
| Malaysia | 30% | 1.00 | 5.00 | 30% | 5% | 80% |
| Paraguay | 30% | 1.00 | 5.00 | 30% | 5% | 80% |
| Peru | 40% | 1.00 | 5.00 | 30% | 5% | 80% |
| Philippines | 30% | 1.00 | 5.00 | 30% | 5% | 80% |
| Thailand | 40% | 1.00 | 5.00 | 30% | 5% | 80% |
| Venezuela | 30% | 1.00 | 5.00 | 30% | 5% | 80% |
| Vietnam | 30% | 1.00 | 5.00 | 30% | 5% | 80% |

**Source**: Expert consultations with IITA, CIAT, and NARS scientists in Africa, LAC, and Asia.

Table 8: Processing technologies for value addition

| **Country** | **Maximum Adoption Rate** | **Research Lag**  **(years)** | **Adoption Lag**  **(years)** | **Yield Increase** | **Input Cost Change** | **Probability of Success** |
| --- | --- | --- | --- | --- | --- | --- |
| Angola | 20% | 8.00 | 12.00 | 25% | 0% | 50% |
| Benin | 20% | 5.00 | 12.00 | 25% | 0% | 50% |
| Burkina Faso | 20% | 8.00 | 12.00 | 25% | 0% | 50% |
| Burundi | 20% | 8.00 | 12.00 | 25% | 0% | 50% |
| Cameroon | 20% | 5.00 | 12.00 | 25% | 0% | 50% |
| Chad | 20% | 8.00 | 12.00 | 25% | 0% | 50% |
| Congo | 20% | 8.00 | 12.00 | 25% | 0% | 50% |
| Cote d’Ivoire | 20% | 8.00 | 12.00 | 25% | 0% | 50% |
| DRC | 20% | 5.00 | 12.00 | 25% | 0% | 50% |
| Ghana | 20% | 3.00 | 12.00 | 25% | 0% | 50% |
| Guinea | 20% | 8.00 | 12.00 | 25% | 0% | 50% |
| Kenya | 20% | 5.00 | 12.00 | 25% | 0% | 50% |
| Liberia | 20% | 8.00 | 12.00 | 25% | 0% | 50% |
| Madagascar | 20% | 8.00 | 12.00 | 25% | 0% | 50% |
| Malawi | 20% | 5.00 | 12.00 | 25% | 0% | 50% |
| Mozambique | 20% | 5.00 | 12.00 | 25% | 0% | 50% |
| Nigeria | 20% | 3.00 | 12.00 | 25% | 0% | 50% |
| Rwanda | 20% | 8.00 | 12.00 | 25% | 0% | 50% |
| Senegal | 20% | 8.00 | 12.00 | 25% | 0% | 50% |
| Sierra Leone | 20% | 8.00 | 12.00 | 25% | 0% | 50% |
| Togo | 20% | 5.00 | 12.00 | 25% | 0% | 50% |
| Uganda | 20% | 3.00 | 12.00 | 25% | 0% | 50% |
| Tanzania | 20% | 5.00 | 12.00 | 25% | 0% | 50% |
| Zambia | 20% | 5.00 | 12.00 | 25% | 0% | 50% |
| Argentina | 16% | 2.00 | 8.00 | 19% | 0% | 80% |
| Bolivia | 18% | 2.00 | 8.00 | 19% | 0% | 80% |
| Brazil | 33% | 2.00 | 8.00 | 22% | 0% | 80% |
| Cambodia | 12% | 2.00 | 8.00 | 20% | 0% | 80% |
| China | 34% | 2.00 | 8.00 | 23% | 0% | 80% |
| Colombia | 30% | 4.00 | 8.00 | 20% | 0% | 80% |
| Costa Rica | 32% | 2.00 | 8.00 | 22% | 0% | 80% |
| Cuba | 23% | 2.00 | 8.00 | 16% | 0% | 80% |
| Ecuador | 25% | 2.00 | 8.00 | 15% | 0% | 80% |
| Haiti | 10% | 2.00 | 8.00 | 16% | 0% | 80% |
| India | 27% | 2.00 | 8.00 | 35% | 0% | 80% |
| Indonesia | 31% | 2.00 | 8.00 | 25% | 0% | 80% |
| Jamaica | 19% | 2.00 | 8.00 | 25% | 0% | 80% |
| Laos | 20% | 2.00 | 8.00 | 25% | 0% | 80% |
| Malaysia | 30% | 2.00 | 8.00 | 24% | 0% | 80% |
| Paraguay | 14% | 2.00 | 8.00 | 22% | 0% | 80% |
| Peru | 20% | 2.00 | 8.00 | 20% | 0% | 80% |
| Philippines | 20% | 2.00 | 8.00 | 19% | 0% | 80% |
| Thailand | 30% | 2.00 | 8.00 | 27% | 0% | 80% |
| Venezuela | 12% | 2.00 | 8.00 | 21% | 0% | 80% |
| Vietnam | 40% | 2.00 | 8.00 | 24% | 0% | 80% |

**Source**: Expert consultations with IITA, CIAT, and NARS scientists in Africa, LAC, and Asia.

Table 9: Strategies to prevent introduction of exotic pests and diseases

| **Country** | **Maximum Adoption Rate** | **Research Lag**  **(years)** | **Adoption Lag**  **(years)** | **Yield Increase** | **Input Cost Change** | **Probability of Success** |
| --- | --- | --- | --- | --- | --- | --- |
| Argentina | 10% | 5.00 | 10.00 | 0% | -10% | 50% |
| Bolivia | 10% | 5.00 | 10.00 | 0% | -10% | 50% |
| Brazil | 25% | 5.00 | 10.00 | 0% | -18% | 50% |
| Cambodia | 50% | 5.00 | 10.00 | 0% | -30% | 50% |
| China | 50% | 5.00 | 10.00 | 0% | -30% | 50% |
| Colombia | 10% | 5.00 | 10.00 | 0% | -10% | 50% |
| Costa Rica | 40% | 5.00 | 10.00 | 0% | -25% | 50% |
| Cuba | 40% | 5.00 | 10.00 | 0% | -25% | 50% |
| Ecuador | 10% | 5.00 | 10.00 | 0% | -10% | 50% |
| Haiti | 40% | 5.00 | 10.00 | 0% | -25% | 50% |
| India | 60% | 5.00 | 10.00 | 0% | -35% | 50% |
| Indonesia | 60% | 5.00 | 10.00 | 0% | -35% | 50% |
| Jamaica | 40% | 5.00 | 10.00 | 0% | -25% | 50% |
| Laos | 50% | 5.00 | 10.00 | 0% | -30% | 50% |
| Malaysia | 50% | 5.00 | 10.00 | 0% | -30% | 50% |
| Paraguay | 10% | 5.00 | 10.00 | 0% | -10% | 50% |
| Peru | 10% | 5.00 | 10.00 | 0% | -10% | 50% |
| Philippines | 50% | 5.00 | 10.00 | 0% | -30% | 50% |
| Thailand | 20% | 5.00 | 10.00 | 0% | -15% | 50% |
| Venezuela | 10% | 5.00 | 10.00 | 0% | -10% | 50% |
| Vietnam | 60% | 5.00 | 10.00 | 0% | -35% | 50% |

**Source**: Expert consultations with CIAT and NARS scientists in LAC and Asia.

Table 10: High-yielding varieties tolerant to cold weather and frost

| **Country** | **Maximum Adoption Rate** | **Research Lag**  **(years)** | **Adoption Lag**  **(years)** | **Yield Increase** | **Input Cost Change** | **Probability of Success** |
| --- | --- | --- | --- | --- | --- | --- |
| Argentina | 100% | 8.00 | 12.00 | 20% | 0% | 50% |
| Brazil | 20% | 8.00 | 12.00 | 20% | 0% | 50% |
| China | 50% | 8.00 | 12.00 | 20% | 0% | 50% |
| Colombia | 10% | 8.00 | 12.00 | 20% | 0% | 50% |
| Vietnam | 20% | 8.00 | 12.00 | 20% | 0% | 50% |

**Source**: Expert consultations with CIAT and NARS scientists in LAC and Asia.
